# Role of the Tumor Microenvironment in Regulating Pancreatic Cancer Therapy Resistance

**DOI:** 10.3390/cells11192952

**Published:** 2022-09-21

**Authors:** Daiyong Deng, Riya Patel, Cheng-Yao Chiang, Pingping Hou

**Affiliations:** 1 Center for Cell Signaling, Rutgers New Jersey Medical School, Newark, NJ 07103, USA; 2 Department of Microbiology, Biochemistry and Molecular Genetics, Rutgers New Jersey Medical School, Newark, NJ 07103, USA; 3 Rutgers Cancer Institute of New Jersey, New Brunswick, NJ 08903, USA

**Keywords:** pancreatic cancer, therapy resistance, tumor microenvironment

## Abstract

Pancreatic cancer has a notoriously poor prognosis, exhibits persistent drug resistance, and lacks a cure. Unique features of the pancreatic tumor microenvironment exacerbate tumorigenesis, metastasis, and therapy resistance. Recent studies emphasize the importance of exploiting cells in the tumor microenvironment to thwart cancers. In this review, we summarize the hallmarks of the multifaceted pancreatic tumor microenvironment, notably pancreatic stellate cells, tumor-associated fibroblasts, macrophages, and neutrophils, in the regulation of chemo-, radio-, immuno-, and targeted therapy resistance in pancreatic cancer. The molecular insight will facilitate the development of novel therapeutics against pancreatic cancer.

## 1. Introduction

Pancreatic ductal adenocarcinoma (PDAC) is a lethal form of pancreatic cancer with an average 5-year survival rate of 11.5% from 2012 to 2018, according to recent data provided by Surveillance, Epidemiology, and End Results (SEER). In 2022, pancreatic cancer accounts for 3% of all cases and 8% of all deaths across cancer types, making it one of the top-five most life-threatening cancers. PDAC is positively correlated with age, having a median diagnosis age of 68 years; however, no sex preference has been determined [1]. Due to the lack of cancer-specific symptoms and prognosis biomarkers, most patients have non-resectable spread tumors at the time of diagnosis. Though PDAC typically forms at the head of the pancreas, where the stomach and the duodenum join and the site of chronic pancreatitis, the underlying mechanisms are still not fully understood.

Risk factors of PDAC include chronic pancreatitis, obesity, tobacco use, type 2 diabetes, and inherited genetic alternations such as mutations in tumor suppressor genes *STK11, BRCA1, BRCA2, CDKN2A,* and genes regulating DNA damage response and DNA repair [2]. More than 90% of PDAC patients have oncogenic *KRAS* mutations. Specifically, KRAS^G12D^ mutation is the most dominant oncogenic mutation and is present in approximately 40% of PDAC cases [3], promoting pancreatic tumorigenesis and maintaining tumor growth [4]. *KRAS* regulates almost all hallmarks of pancreatic cancer, especially activating essential signaling pathways for proliferation and survival, rewiring anabolism, and suppressing immune response in the tumor microenvironment (TME) [5]. Mouse model studies suggest that mutant *KRAS* alone can induce replication stress in pancreatic epithelial cells and is not sufficient to drive malignancy. Other factors are required to promote PDAC development, including chronic inflammation (pancreatitis) and loss of tumor suppressor genes, among which *CDKN2A, CDKN2B, TP53,* and *SMAD4* are frequently detected in PDAC accompanied by KRAS mutations [6]. According to integrated genomic analysis, PDAC can be generally classified into four different subtypes: squamous/quasi-mesenchymal/basal-like, pancreas progenitor/classical, immunogenic, and aberrantly differentiated endocrine exocrine (ADEX)/exocrine-like subtypes [7]. The squamous subtype is associated with the poorest overall outcomes and is least dependent on KRAS signaling.

In this review, we summarize therapeutic options for PDAC and current challenges, discuss the hallmarks of the pancreatic TME and the role of TME in regulating therapy response, and provide outlook on future directions to address the difficulties associated with therapy resistance.

## 2. Therapeutic Options for PDAC Patients

Despite great progress in immunotherapy and targeted therapy of other cancer types, PDAC patients receive minimum benefit due to the lack of efficacy and unacceptable toxicity of KRAS signaling inhibitors (e.g., receptor tyrosine kinase (RTK), mitogen-activated protein kinase kinase (MEK) inhibitors) as well as the immune suppressive TME. Surgical resection is currently the only curative modality for PDAC. However, less than 20% of patients are eligible for surgical treatment because of poor health conditions or distant metastasis. Chemotherapy (e.g., gemcitabine/capecitabine, FOLFIRINOX) or/and radiotherapy remain the standard-of-care, which have exhibited improved long-term outcomes in PDAC patients [2]. Recently, pan-RAS and KRAS^G12D^ inhibitors have been developed and are being actively evaluated in pre-clinical settings [5]. A KRAS^G12D^ inhibitor MRTX1133 dramatically suppressed PDAC growth in pre-clinical xenograft models [8], bringing new hope to PDAC patients. Besides chemical inhibitors, new therapeutics targeting KRAS mutant cancers include exosome-delivered KRAS siRNA (iExosome), mRNA vaccines, anti-KRAS T cell transfer, and cell-permeable RAS antibodies [5]. Of these therapeutics, T cell therapy and the iExosome method are currently in phase I clinical trials now. However, therapy resistance remains intractable, and both tumor cell-intrinsic and -extrinsic mechanisms have been identified. Clinical results of KRAS^G12C^ inhibitors suggest that targeting KRAS rewires TME from immune suppression to pro-inflammation, and combination therapy is required to enhance tumoricidal effect and prevent adaptive resistance. 

## 3. Hallmarks of the Pancreatic Tumor Microenvironment

**Figure 1:**
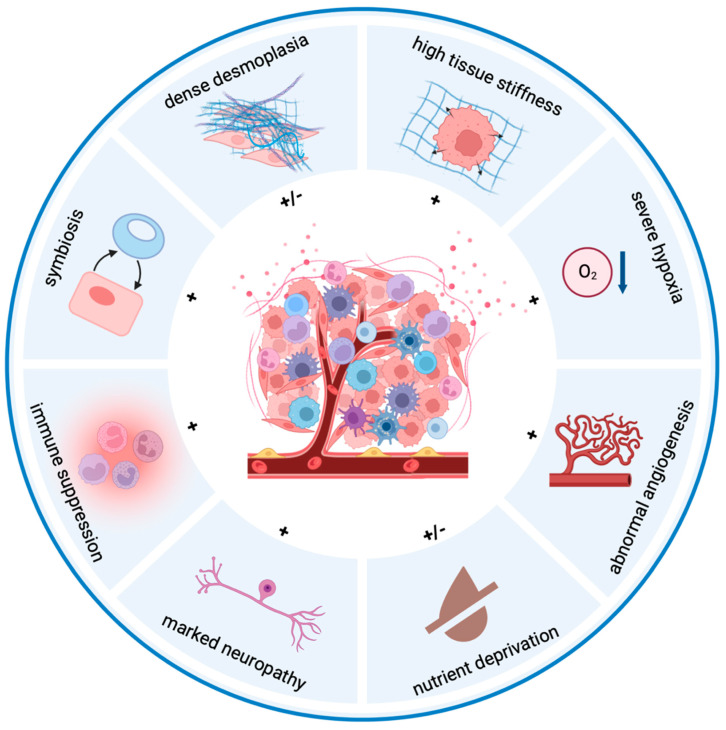
**Hallmarks of the pancreatic tumor microenvironment.** The unique features of the pancreatic tumor microenvironment compared to other solid cancers are summarized. Pancreatic cancer is composed of abundant stroma (fibroblasts, stellate cells, neuronal cells, endothelial cells, etc.) and immune suppressive cells. The dense extracellular matrix results in high tissue stiffness and severe hypoxia. The hyperproliferative tumor cells and active stroma deprive essential metabolites in situ, and the hostile milieu further exacerbates the exclusion of cytotoxic immune cells. Moreover, tumor cells benefit from cells of the tumor microenvironment to progress, migrate and escape from therapy. “+”, pro-tumor hallmarks; “−“, anti-tumor hallmarks.

Malignant cells rewire the microenvironment. The main cell populations of the PDAC TME consist of pancreatic stellate cells (PSCs), cancer-associated fibroblasts (CAFs), and myeloid cells, as well as regulatory T cells, B cells, and neuronal cells [9]. They can either support or block tumor development and maintenance depending on their received signals, and the sum of their effects results in unique hallmarks of the pancreatic TME: dense desmoplasia, high tissue stiffness, severe hypoxia, abnormal angiogenesis, nutrient deprivation, marked neuropathy, extensive immune suppression, and symbiosis (Figure 1). In detail, PSCs and CAFs produce abundant extracellular matrix (ECM) molecules such as proteoglycans, collagens, and fibronectin [10], leading to fibrosis and tissue rigidity and thus contributing to hypoxia and supporting tumor progression and metastasis [11,12]. Hypoxia rewires tumor metabolism, promotes tumor proliferation, survival, and epithelial-to-mesenchymal transition (EMT), and contributes to immune suppression [13]. Increased tissue stiffness drives PDAC invasion [14]. However, distinct subtypes of CAFs may exhibit opposite roles, such as that α-smooth muscle actin-positive (αSMA+) myofibroblasts restrain tumor growth partially by preventing the infiltration of immune suppressive cells [15,16].

Immune cells are another major component in the pancreatic TME. The major myeloid cell populations include macrophages, neutrophils, monocytes, and dendritic cells (DCs). In response to inflammation, pancreas infiltrated macrophages drive the acinar-to-ductal metaplasia transdifferentiation by producing inflammatory cytokines C-C motif chemokine ligand 5 (CCL5) and tumor necrosis factor α (TNFα) to activate nuclear factor kappa B (NFκB) signaling pathway, resulting in ECM remodeling and epithelial cell transformation [17]. In PDAC, tumor-associated macrophages (TAMs), tumor-associated neutrophils (TANs), and myeloid-derived suppressor cells (MDSCs) are dominant while DCs are sparse, preventing cytotoxic T cells and natural killer (NK) cells from penetrating the tumor [18,19]. In addition, regulatory CD4+ Foxp3+ T cells (Tregs) and regulatory B cells accumulate in advanced PDAC to promote immune tolerance by secreting inhibitory cytokines such as interleukin (IL)-10, transforming growth factor β (TGFβ) and IL-35 [10,20,21]. Tregs also interact with DCs and suppress their costimulatory ligand expression to restrain CD8+ T cell activation [22]. Surprisingly, ablation of Tregs in a PDAC mouse model accelerates tumor progression [23]. Mechanistic analysis reveals that Treg depletion causes CAF reprogramming by loss of tumor-restraining αSMA+ fibroblasts and gain of C-C motif chemokine receptor 1 (CCR1) ligand expression, recruiting myeloid cells to restore the immune suppression. Moreover, γδT cells constitute about 40% tumor infiltrating T cells in human PDAC, which are considered major sources of immune suppressive checkpoint ligands [24]. 

KRAS can increase granulocyte-macrophage colony-stimulating factor (GM-CSF) expression in mouse pancreatic ductal epithelial cells, and GM-CSF upregulation is also observed in human pancreatic neoplasia lesions [25]. GM-CSF recruits Gr1+ myeloid cell infiltration, and their pro-tumor activity is mediated by CD8+ T cell suppression [25,26]. In addition, cancer-associated mesenchymal stem cells (MSCs), rather than normal pancreas MSCs, stimulate alternative polarization of macrophages [27]. A recent study reveals that lysine demethylase 3A (KDM3A) is an epigenetic regulator of the immunotherapy response whose effect is mediated by transcription factors KLF5 and SMAD4 in PDAC [28]. Epidermal growth factor receptor (EGFR) is their downstream factor, and its inhibition facilitates CD8+ T cell infiltration, reduces myeloid cells, and sensitizes pancreatic tumors to combination immunotherapy (CD40 agonist, programmed cell death protein 1 (PD-1) and cytotoxic T-lymphocyte-associated protein 4 (CTLA-4) blockade).

The pancreatic TME is highly innervated [29]. In a clinical study, 100% of PDAC patients (132/132) had neural invasion [30]. Tumor cells secrete neurotrophins to promote neuron infiltration and stimulate neuron growth [31]. On the other hand, premalignant pancreatic cells are prone to invade the spinal cord along sensory neurons. Ablation of sensory neurons in PDAC mouse models blocks the inflammatory signal transduction from pancreatic neoplasia to the central nervous system and hinders disease progression [32,33]. Sensory neurons produce stress molecules such as catecholamines that bind to β-adrenergic receptors on PDAC cells to promote tumorigenesis and tumor growth [34,35]. Upon pathway activation, PDAC cells increase nerve growth factor (NGF) expression, leading to perineural invasion and enlarged intratumoral nerves [35]. Blockage of the NGF/neurotrophic receptor tyrosine kinase 1 (Trk) pathway impairs tumor growth, prolongs mouse survival, and enhances tumoricidal effect of gemcitabine in spontaneous PDAC mouse models [35]. While subdiaphragmatic vagotomy accelerates PDAC progression, systemic administration of bethanechol, a muscarinic agonist, impairs tumor growth and prolongs mouse survival [36]. Mechanism dissection reveals that the cholinergic receptor muscarinic 1 (CHRM1) receptor expressed by tumor cells is responsible for the cholinergic suppressive effect via modulating mitogen-activated protein kinases (MAPK) and phosphatidylinositol-3-kinase (PI3K)-AKT pathways. Moreover, a recent study discovered that neurons nourish PDAC cells with serine to facilitate protein translation. Increased NGF production, in turn, exacerbates tumor innervation [37].

The symbiotic relationship between cancer cells and cells of the TME supports PDAC growth. KRAS promotes the secretion of sonic hedgehog protein (SHH) by cancer cells, which can induce extensive proteomic changes in PSCs [38]. The changes include the upregulation of ECM components such as matrix metalloproteinases (MMPs) and collagens, suggesting that KRAS is a driver of tumor desmoplasia. SHH also elevates growth factors insulin-like growth factor 1 (IGF1) and growth arrest-specific 6 (GAS6) that reciprocally activate IGF1R/AXL-AKT signaling pathway and increase spare mitochondrial capability in PDAC cells. In addition, fibroblast growth factor 1 (FGF1) secreted by CAFs is essential for paracrine MYC activation and protein stability coordinately with pancreatic tumor cell-autonomous signals [39]. Moreover, KRAS upregulates type I cytokine receptor complexes (IL2rγ-IL4rα and IL2rγ-IL13rα1) in pancreatic neoplasia. Tumor-infiltrated T helper 2 (Th2) cells produce IL-4 and IL-13 to activate Janus kinase 1 (JAK1)- signal transducer and activator of transcription 6 (STAT6)-MYC axis and enhance glycolysis, thus promoting tumorigenesis [40]. The accumulation of Th2 cells in TME needs intercellular cooperation. TNFα and IL-1β from tumor cells enable activation of CAFs to produce thymic stromal lymphopoietin (TSLP), which promotes Th2 polarization via DC conditioning [41]. Monocyte-recruited basophils stabilize the Th2 phenotype in pancreatic tumor-draining lymph nodes by releasing IL-4 [42]. Finally, Th2 cells are recruited into the TME in response to Th2-attracting chemokines secreted by tumor cells [41]. 

Rapid tumor growth causes nutrient deprivation in the TME. Thus, the reciprocal intercellular interaction is critical for nutrient exchange [43]. PDAC cells are addict to glucose and glutamine [4,44], and circulating lactate is a primary carbon source for the tricarboxylic acid (TCA) cycle in fasted mice [45]. To fulfill the high demand of amino acids, PDAC elevates macropinocytosis via KRAS to scavenge macromolecules from surroundings [46,47]. In a biological process called reverse Warburg effect [48], tumor cells stimulate CAFs to secrete metabolic intermediates such as pyruvate and lactate, which are reciprocally taken in by tumor cells for ATP production. Moreover, alanine secreted by activated PSCs serves as an alternative carbon source for PDAC to fuel TCA cycle and biosynthesis of non-essential amino acids (NEAA) and lipids [49]. Notably, the secretion of alanine by PSCs requires cancer cell stimulated autophagy activity. Despite PDAC cells’ ability to exploit stroma cells to adapt to the nutrient-deprived condition, CD8+ T cells usually exhibit impaired function and proliferation [50]. Specifically, MDSCs and TAMs express high levels of arginase and nitric oxide synthase, which consume arginine in the pancreatic TME, a critical amino acid for T cell activation [51].

The physicochemical features and intercellular crosstalk in the TME not only regulate pancreatic tumorigenesis, tumor maintenance and metastasis, but they are also critical elements determining tumor responses to cancer therapies. Hypoxia, a nonnegligible resistance inducer, has been extensively reviewed elsewhere [52,53]. Thus, we focus on the interplay between cancer cell and non-cancer cell counterparts that prevents PDAC from apoptosis upon therapeutic treatments. Accumulated evidence suggests that PSCs, CAFs, TAMs and TANs/MDSCs are major players of therapy resistance, and their multifaced roles are discussed in the following sections.

## 4. Pancreatic Stellate Cells and Therapy Resistance

Desmoplasia, a hallmark of pancreatic cancer, is mainly attributed to pro-fibrogenic PSCs. The origin of PSCs is still debated despite bone marrow, monocytes, and mesenchymal cells being considered the source of PSCs [54,55]. In contrast to fibroblast cells, PSCs have a star-like shape, express glial fibrillary acidic protein (GFAP), and store cytoplasmic vitamin A-containing lipid droplets. Upon stimulation by pathogenic factors such as reactive oxygen species (ROS), hypoxia, cytokines, growth factors and toxins, PSCs are transdifferentiated from a quiescent to an activated myofibroblast-like state. This results in the loss of PSC-specific markers and gain of α-SMA expression, accompanied by production of abundant ECM proteins, MMPs and autocrine cytokines, promoting fibrosis and sustaining self-activation. 

The severe desmoplastic and fibroinflammatory phenotype of PDAC facilitates therapy resistance. Preventing PSC activation by depletion of integrin α5 (ITGA5) abolishes desmoplasia in PDAC xenograft models, increases vasculature maturation, and sensitizes cancer cells to gemcitabine [56]. Reprogramming of activated PSCs into the quiescent state by vitamin D receptor ligand results in reduced inflammation and fibrosis and increased intratumoral gemcitabine in vivo, enhancing chemotherapy sensitivity [57]. In addition, chemotherapeutics is sequestered in the stroma counterpart, preventing successful delivery to cancer cells [58,59]. In regard to reciprocal connection, autocrine periosin (osteoblast-specific factor 2) stimulates PSCs to express collagen I, fibronectin, and transforming growth factors, leading to PDAC cell chemoresistance in vitro [60,61]. Among the ECM proteins, collagen I and fibronectin increase ERK1/2 phosphorylation, attenuate gemcitabine-induced cell cycle arrest, and promote cell proliferation [62,63,64]. Paracrine growth factors from activated PSCs including IGF1 and IGF2, leukemia inhibitory factor (LIF), and hepatocyte growth factor (HGF), act on PDAC cells to prevent gemcitabine-induced cell death via hyperactivation of mitogenic and survival signaling pathways [65,66,67]. Specifically, IGF and HGF upregulate the PI3K/AKT pathway and are associated with EMT. Besides the LIFR/STAT3 pathway, canonical Wnt and hippo pathway genes are elevated by LIF. Though both IL-6 and LIF are extensively expressed by PSCs and activate STAT3 signaling, only LIF expression is positively correlated with disease progression, and LIF blockade sensitizes PDAC cells to chemotherapy in mouse models. Upon activation by TGF-β, PSCs are the source of ECM protein cysteine-rich angiogenic inducer 61 (CYR61) which attenuates gemcitabine uptake in PDAC cells by suppressing the expression of nucleoside transporters equilibrative nucleoside transporter 1 (ENT1) and concentrative nucleoside transporter 1 (CNT3) [68]. Moreover, metabolite deoxycytidine released by PSCs competes with gemcitabine for deoxycytidine kinase (dCK)-mediated phosphorylation, protecting PDAC cells from gemcitabine toxicity [69]. 

**Figure 2:**
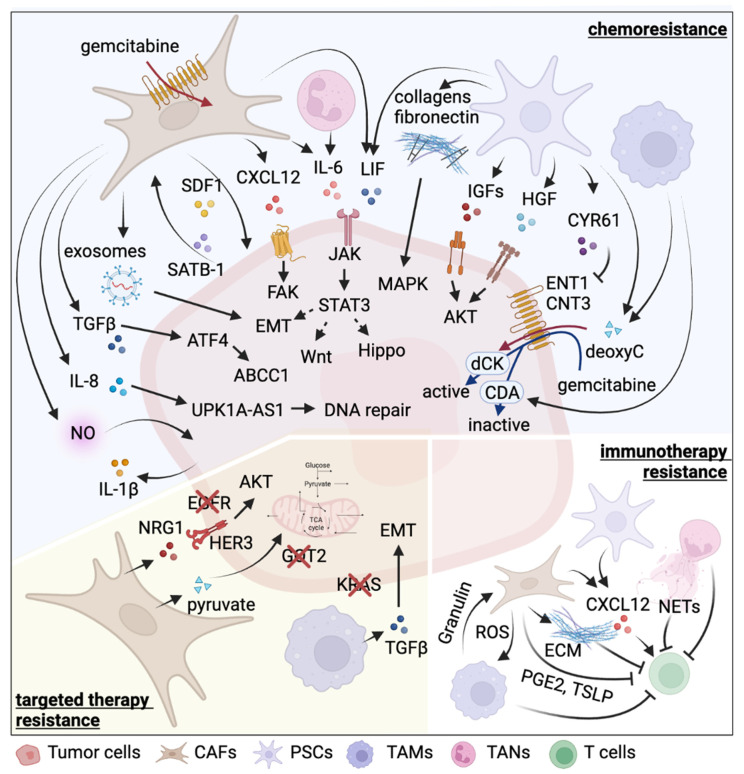
**Therapy resistance mechanisms driven by the tumor microenvironment in pancreatic cancer.** Role of the TME in chemoresistance are extensively studied, while its function in regulating tumor response to novel therapeutics needs further investigation. For chemoresistance mechanisms, the TME mainly provides growth factors to promote tumor cell survival and decreases the uptake or inactivate chemo drugs. For targeted therapy resistance, the TME usually nourishes tumor cells by activating parallel or alternative pathways. For immunotherapy resistance, the TME not only forms physical barriers to prevent the cytotoxic immune cell infiltration, but also produces immune suppressive factors to inactivate them. CDA, cytidine deaminase; dCK, deoxycytidine kinase; deoxyC, deoxycytidine.

It is expected that PSCs contribute to immune suppression. Activated PSCs not only recruit CD8+ T cells via chemokine ligand 12 (CXCL12) chemotaxis and exclude them from juxtatumoral region [70], but they also suppress T cell proliferation and induce Th2 differentiation by secreting Galectin-1 [71]. IL-6 secreted by PSCs promotes MDSC differentiation via STAT3 activation [72]. Taking all these effects into account, PSCs seem to be a promising therapeutic target to sensitize PDAC cells to conventional modalities, despite the controversial clinical results on the potential of monotherapy [55]. The lack of efficacy of targeting CAFs alone may be due to the debated roles of PSCs in restraining and nourishing tumor cells. Thus, rather than cell depletion, strategies that reprogram activated PSCs into the quiescent state are likely to circumvent potential negative effects and synergize with other therapeutics to impair tumor growth [57,73,74]. A summary of therapy resistance mechanisms mediated by intercellular interactions is listed in Table 1 and illustrated in Figure 2.

## 5. Cancer-Associated Fibroblasts and Therapy Resistance

CAFs are spindle-shaped, non-neoplastic cells located in the TME. Pancreatic fibroblasts and PSCs are considered two different cell populations due to their distinct morphologies and gene expression [54]. However, the composition of CAFs is complicated, including tissue-resident fibroblasts, activated PSCs, adipocytes, mesenchymal stem cells, transitioned mesothelial cells, epithelial cells, endothelial cells, transformed hematopoietic stem cells, and circulating bone marrow cells [108]. Utilizing single-cell assays to characterize the heterogeneity of CAFs in PDAC, three functionally distinct CAFs have been identified: myofibrobastic CAFs (myCAFs), inflammatory CAFs (iCAFs), and antigen-presenting CAFs (apCAFs) [109,110,111]. The myCAFs are adjacent to tumor cells, express high αSMA, and produce desmoplastic stroma. In contrast, the iCAFs are relatively distant from the neoplastic region, lack αSMA expression, and secrete inflammatory cytokines such as IL-6, IL-11, and LIF. The recently characterized apCAFs express major histocompatibility complex (MHC II) and CD74 and are capable of presenting antigens to CD4^+^ T cells, despite the low efficiency in comparison to professional antigen-presenting cells (APCs). The apCAFs may be orientated from mesothelial cells induced by IL-1 and TGFβ [112]. CD105 is another marker that can separate two discrete CAF populations [111]. While the CD105^+^ CAFs promote tumor growth, the CD105^-^ CAFs attenuate tumor growth. Notably, CD105 expression is not restricted to either previous CAF subpopulation, but apCAFs are CD105^-^, which may explain the tumor suppressive effect.

CAFs play a major role in therapy resistance. In general, CAFs produce a dense ECM and increase tissue stiffness to form a physical barrier in TME that prevents drug delivery. The dense stroma and poor vascularization cause severe hypoxia. Activation of PI3K-Akt, NF-κB, and Notch pathways partially explains chemoresistance induced by hypoxia [113,114]. Hypoxia-inducible factor 1 (HIF-1) upregulates ATP-binding cassette subfamily G member 2 (ABCG2) in PDAC to promote gemcitabine efflux [115]. In addition, neutral amino acid transporter B(0) (SLC1A5) can be induced by HIF-2α to promote glutamine-dependent ATP production and glutathione synthesis, conferring gemcitabine resistance in PDAC [116]. Depletion of HIF-2α in CAFs significantly reduces M2-like TAMs polarization and recruitment in pancreatic TME, sensitizing PDAC to immune checkpoint blockade [117]. A detailed summary of hypoxia-driven cancer progression and therapy resistance can be found in recent reviews [13,52,53].

CAFs also alter the drug metabolism to reduce the tumor response [118]. Observed gemcitabine accumulation in CAFs (despite intrinsic resistance) suggests the contribution of CAFs to scavenging chemo drugs [75]. Another proposed mechanism reveals that CAFs elevate exosome secretion upon gemcitabine exposure, which stimulates EMT-TF *Snai1* expression in recipient tumor cells and results in drug resistance [76]. Recent studies have identified several novel reciprocal mechanisms. Specifically, the upregulation of circular RNA circFARP1 in CAFs enhances LIF secretion, which induces tumor cell stemness and chemoresistance in PDAC via CAV1/miR-660-3p axis in vitro [77]. IL-8, secreted by CAFs, upregulates long non-coding RNA (lncRNA) UPK1A-AS1 expression in tumor cells, facilitates DNA double-strand break (DSB) repair, and confers chemoresistance in PDAC xenograft models [82]. In addition, CAFs induce activating transcription factor 4 (ATF4) expression in PDAC cells via canonical TGFβ pathway activation. The elevated ATF4 is associated with poor prognosis and drives gemcitabine resistance via upregulation of ATP-binding cassette (ABC) transporter ABCC1 in mouse models [78]. Another reciprocal feedback between CAFs and PDAC cells contributes to chemoresistance is stromal cell-derived factor 1 (SDF-1)/SATB-1 axis. CAFs secretes SDF-1 to induce SATB-1 production in tumor cells, and CATB-1 maintains CAF activation [79]. IL-6-JAK-STAT3 pathway is another key mediator of chemoresistance in PDAC. The combination of gemcitabine and IL6R blockade suppresses tumor growth and prolongs the mouse overall survival [80]. In addition, CAFs nourish CXCR4+ pancreatic cancer stem cells by producing its ligand CXCL12, promoting tumor cell growth and gemcitabine resistance via activation of FAK, AKT and ERK signaling pathways [81]. Moreover, nitric oxide (NO) released by the fibroblasts can induce IL-1β secretion in PDAC cells, which binds to its receptor expressed on tumor cells to confer chemoresistance in a paracrine manner [83]. Beside chemoresistance, the inhibition of CAF-PDAC cell crosstalk by interrupting neuregulin-1 (NRG-1)- Erb-B2 receptor tyrosine kinase 3 (ErbB3) axis overcomes EGFR targeted therapy resistance in PDAC pre-clinical models [84,85].

CAFs play an immunosuppressive role in PDAC [119]. The dense collagen network acts as a physical barrier to prevent chemoattractant T cell migration [86]. CAFs induce M2 macrophage polarization by producing M-CSF to elevate ROS in monocytes [87]. Depletion of fibroblast activation protein-positive (FAP+) stroma cells enables immunological control of tumor growth by IFNγ and TNFα [88]. Specifically, CXCL12 is dominantly secreted by CAFs and excludes T cells from the tumor region. Inhibition of corresponding receptor CXCR4 in combination with PD-L1 immune checkpoint blockade synergistically impedes tumor growth [89]. Prostaglandin E2 (PGE2) is another reported mediator of T cell suppression by CAFs; blocking it restores T cell proliferation and decreases the expression of T cell exhausted markers [90]. In addition, the Th2 response is associated with reduced patient survival, and CAFs favor Th2 cell polarization and recruitment [41]. Upon stimulation by TNFα and IL-1β from PDAC cells, CAFs secrete thymic stromal lymphopoietin (TSLP) to activate dendritic cells via receptor interaction and induce the Th2 phenotype.

Furthermore, CAFs render the metabolic dependency of PDAC [120]. Depletion of glutamic-oxaloacetic transaminase 2 (GOT2) breaks the redox balance and inhibits PDAC cell growth in vitro, but it has little effect on tumor growth in vivo [91]. Metabolic analysis reveals that CAFs provide tumor cells with pyruvate to overcome GOT2 dependency. Interestingly, blockage of pyruvate importation or pyruvate-to-lactate reduction cannot impair GOT2-depleted tumor growth, indicating hyperdynamic metabolic crosstalk in TME. Another study utilizing optical imaging to assess the redox status of PDAC cells suggests that CAFs aid tumor cells in surviving in a more oxidative state [92].

CAFs are the dominant component in the PDAC TME with diverse functions in tumorigenesis and therapy resistance. Given the hyper-heterogeneity, a delicate design to specifically restrict pro-tumor roles is required for CAF-targeted therapy. Current strategies include direct targeting of CAFs, interfering with intercellular crosstalk, ECM disruption, CAF inactivation, and reprogramming [108,118,121]. Depletion of FAP+ CAFs by antibody-drug conjugate (ADC) OMTX705 combined with gemcitabine achieved durable tumor regression for more than 90 days in a PDAC PDX model [122]. This treatment is well tolerated, suggesting desmoplastic stroma targeting is a compelling therapeutic strategy. T cells redirected by anti-FAP chimeric antigen receptor (CAR) impair tumor growth in lung cancer models [123]. Whether it works for PDAC and synergizes with other modalities requires further investigation. Clinical trials evaluating CAF-targeted therapy are listed in Table 2.

## 6. Tumor-Associated Macrophages and Therapy Resistance

Macrophages regulate tissue development and maintain tissue homeostasis [124]. They are tissue-resident or infiltrated from circulating bone marrow-derived monocytes. Due to their hyperplastic nature, macrophages are polarized distinctly depending on stimuli and are mainly classified into five subtypes: pro-inflammatory M1 and immune-tolerant M2 (M2a, M2b, M2c, and M2d). Macrophages are phagocytes that can engulf and digest foreign pathogens and apoptotic cells regardless of polarization status. In addition, macrophages are professional APCs that process and present antigens for T cell recognition. Macrophages are abundant in the pancreatic TME, and M2 TAMs are correlated with poor overall survival [125]. However, M1 and M2 definitions could not accurately describe the heterogeneity of TAMs, which is fully reflected by single-cell transcriptional analysis. For example, Tie2+ M2 TAMs are a predictive marker of poor prognosis in multiple cancers, including PDAC, which may regulate angiogenesis via the ang2-Tie2 axis [126,127]. TAMs in PDAC are composed of pancreas resident macrophages orientated from the yolk sac and circulating monocytes [128]. Notably, embryonic progenitor-derived TAMs express more ECM molecules, regulating collagen deposition and fibrosis. In contrast, monocyte-derived TAMs have high cytokine expression and antigen presentation molecules, suggesting a role in modulating cancer immunity. CSF1 signaling is crucial for macrophage differentiation, infiltration, local expansion, and survival [129]. C-C motif chemokine ligand 2 (CCL2)-C-C Motif Chemokine Receptor 2 (CCR2) axis is the major chemoattractant signaling of macrophages. Blockage of either pathway decreases TAM population and impedes tumor growth [130], making them both promising therapeutic targets to limit TAM pro-tumor activities.

TAMs exacerbate desmoplasia, angiogenesis, nutrient deprivation, and immune suppression to promote tumor growth by producing cytokines, chemokines, growth factors, and ECM components [126,131]. Meanwhile, TAMs are a key player in regulating therapy resistance. TAMs accumulate in TME after therapies [95,99,132], contributing to pancreatic tumor recurrence. To confer chemoresistance, TAMs release deoxycytidine via the transcription factor C/EBPδ to interfere with the uptake and metabolism of gemcitabine [93,133]. TAMs stimulate PDAC cells to upregulate cytidine deaminase expression, which eliminates gemcitabine [94]. By accumulating around blood vessels, TAMs promote tumor revascularization via secreting VEGF-A after chemotherapy to support tumor relapse in mouse models [132]. TAMs are also involved in radiotherapy resistance. Whereas the enrichment of CCR2+ macrophages has been observed in PDAC after radiotherapy, neutralizing or genetic depletion of CCL2 improves radiotherapy responses and attenuates tumor growth in mouse models [99]. Upon KRAS targeted therapy, macrophage infiltration dramatically increases in therapy-resistant PDAC tumors in pre-clinical models [95], which are essential and sufficient to drive KRAS bypass. Mechanistically, PADC cells elevate the production of CCL2 chemokine to attract CCR2+ M2-like macrophage infiltration, which reciprocally provides tumor cells with abundant TGFβ to promote KRAS-independent tumor growth. TGFβ is a robust driver of EMT, which is associated with KRAS inhibition resistance in PDAC and lung cancer cells [134,135,136]. TAMs may also induce EMT in PDAC via the secretion of MMP9 [137].

PDAC is irresponsive to immunotherapy. TAMs in peri-tumor regions form a barrier against T cells [100]. Although chemotherapy induces immune suppressive TAMs, these p21^high^ TAMs respond to CD40 agonists [138]. TAMs secrete granulin upon M-CSF stimulation in the TME, and granulin is essential for local fibrosis and exclusion of T cells at the metastatic site in mouse models [96]. Depletion of granulin allows T cell entry in the liver and sensitizes metastatic PDAC tumors to PD-1 blockade. In addition, PDAC upregulates necroptosis complex component receptor-interacting serine/threonine-protein 1 (RIP1) in both tumor epithelial cells and stroma to induce chemokine CXCL1 expression, which attracts macrophage infiltration [97]. Interaction of TAMs and tumor cells via ligation of Mincle and Sin3A-associated protein 130 (SAP130) polarizes TAMs to immune suppressive M2 phenotype, resulting in adaptive immune suppression and tumor progression. Depletion of RIP1 in epithelial cells or TME cells is protective against PDAC. In addition, chemical inhibition of RIP1 reprograms TAMs toward immunogenic M1 via STAT1 activation [98]. Educated M1 TAMs elicit the cytotoxicity of CD8+ T cells and promote Th1 and Th17 differentiation of T helper cells. Combination therapy of RIP1 inhibitor and PD-1 blockade synergistically induces tumor immunity and suppresses PDAC tumor growth in vivo.

In conclusion, the targeting or reprogramming of TAMs is likely to enhance the tumoricidal effect of multiple therapies through disruption of intercellular crosstalk between TAMs and other cell counterparts, leading to remodeling of the pro-tumor to anti-tumor TME. It is noticed that macrophage status is a highly dynamic spectrum and determined by external stimuli; thus, in vivo reprogramming of TAMs may be more challenging than previously expected.

## 7. Tumor-Associated Neutrophils and Therapy Resistance

Neutrophils are the most abundant and short-lived innate immune cells, and they are responsible for mediating the rapid innate host defense against pathogens [139]. They infiltrate solid tumors and have attracted much attention in recent years [140,141,142,143]. It is still challenging to distinguish between TANs and polymorphonuclear MDSCs (PMN-MDSCs) (a.k.a. granulocytic MDSCs (G-MDSCs)) because of the lack of unique markers, while both are considered immune suppressive in most cancer cases. Similar to macrophages, TANs are classified into two major polarization states, anti-tumor N1 and pro-tumor N2 [144]. TGFβ renders the compromised cytotoxicity of TANs via a superoxide-dependent mechanism and promotes the anti-inflammatory N2 phenotype. On the other hand, IFNβ, IFNγ, and GM-CSF have been shown to polarize TANs into a pro-inflammatory, APC-like N1 state [145,146]. In contrast to N1 TANs, which have lobulated and hyper-segmented nuclei and express CD101 marker [147], N2 TANs appear immature with circular and less lobulated nuclei and high levels of CD170 [148]. To note, the phenotype of TANs is more dynamic and heterogeneous than the dichotomized classification, so a mixed state is usually observed. In PDAC, TAN presence is an independent prognosis factor for tumor recurrence and overall survival [149].

By producing ROS and arginase 1 (ARG1), modulating multiple signaling pathways, and forming neutrophil extracellular traps (NETs), TANs not only promote tumor metastasis and angiogenesis but also suppress NK and T cell cytotoxicity and induce immune tolerance [139,141]. Neutrophils express CXCR1 and CXCR2 receptors whose ligands such as CXCL1, 2, 5, and 8 are dramatically upregulated by PDAC cells, and high CXCL5 is positively correlated with poor patient overall survival [102]. In addition, cytokines G-CSF and GM-CSF, rather than M-CSF, regulate neutrophil recruitment, survival, and differentiation, and they are significantly upregulated in PDAC versus a normal pancreas.

Targeting TANs improves conventional therapy response in PDAC. IL6 receptor blockade suppresses STAT3 phosphorylation in both myeloid cells and tumor cells, thus sensitizing tumor cells to gemcitabine [80]. In addition, CXCR2 inhibition prevents compensatory infiltration of CXCR2+ TANs upon CCR2+ TAM depletion in PDAC models, resulting in improved tumoricidal immunity and better response to chemotherapy regimens [102]. In a KRAS-driven sarcoma model, depletion of TANs by anti-Ly6G neutralizing antibody enhances radiotherapy responses [104].

Furthermore, TANs (or G-MDSCs) drive immunotherapy resistance. By multidimensional imaging, G-MDSCs expressing high lectin-like oxidized low-density lipoprotein receptor 1 (LOX-1) and ARG1 are shown to reduce the expression of granzyme B and Ki67 in colocalized T cells [103]. Another recent study reveals that the immunosuppressive role of IL-17 in PDAC is mediated by TANs [101]. Specifically, IL-17 recruits neutrophils in TME and promotes NETs formation. Blockade of IL-17 enables PDAC cells to respond to checkpoint blockade, and it synergizes with PD-1 to impair tumor growth in a CD8+ T cell-dependent manner in pre-clinical models. In addition, there is a positive correlation between NETosis and poor overall survival in PDAC patients. Depletion of CXCR2+ TANs attenuate PDAC metastasis, promotes T cell entry, and sensitizes tumor cells to PD-1 blockade in mice [107]. In a later study dissecting immune heterogeneity of various PDAC subclones, immunotherapy-resistant ones lack T cell infiltration but enrich G-MDSCs [106]. Tumor cells secrete CXCL1 to recruit CXCR2+ G-MDSCs. Depletion of CXCL1 overcomes resistance to combination immunotherapy (anti-CD40 agonist, anti-PD-1 antagonist, and anti-CTLA-4 antagonist) in syngeneic mouse models. Similarly, in a p53R172H mutant PDAC model, neutrophils are recruited by tumor cells via the CXCL2/5–CXCR2 axis. Depletion of TANs increases T cell infiltration and enhances the tumoricidal activity of CD40 agonist and gemcitabine/nab-paclitaxel combination [105]. The lack of T cell activation indicates that the addition of T cell immune checkpoint blockade may further impair tumor growth. Additionally, STAT5 inhibition by lorlatinib blocks tumor-induced granulopoiesis and suppresses neutrophil migration, leading to enhanced immunotherapeutic responses and PDAC regression in vivo [150].

In summary, immune suppression is the dominant role of TANs/G-MDSCs, and they can rewire the TME by autocrine or paracrine mechanisms. Depletion of TANs or MDSCs by CXCR2 inhibitors augments immunotherapy response in several cancer models [151,152]. Whether CXCR2 inhibition can also sensitize PDAC patients to checkpoint blockade needs further clinical investigation. Besides targeting neutrophil recruitment, modalities to repolarize N2 TANs into N1 pro-inflammatory phenotype are in development as well, such as combinations of TGFβ signaling inhibitors and immune checkpoint blockade in clinical trials (Table 2). Survival pathways for TANs, including PI3K gamma/delta (PI3Kγ/δ), are other promising targets to reduce the TAN population.

## 8. Perspectives, Challenges, and Future Directions

The crucial role of the TME in regulating therapy responses has been recognized since the 2000s [13]. Thanks to the development in analysis technologies, scientists can characterize components of the TME, delineate spatiotemporal regulation and demonstrate intercellular interactions at single-cell resolution, and dissect regulatory mechanisms at genetic, epigenetic, transcriptional, protein, and metabolic levels. In contrast to tumor intrinsic therapy resistance, extrinsic mechanisms involve multi-players and complicated crosstalk, increasing difficulties in discovering the authentic driver events. 

The primary challenge arises from the heterogeneity and dynamics of the TME across different PDAC models. Compared to spontaneous tumor models, transplanted tumors generally exhibit relatively homogeneous tumor histology with decreased stroma penetration and immune cell diversity. The difference may not be dramatic, but it still possibly affects the readout and conclusions made on the roles of the TME. However, the human relevance of the mouse TME in spontaneous tumor models needs to be addressed in a higher resolution. The phenotype, physicochemical characteristics, and intercellular spatial relationships of the TME in human PDAC and different mouse PDAC models have not been fully understood and compared yet. Thus, comprehensive single-cell analysis and characterization of hallmarks of the pancreatic TME in comparison of the two species are needed to enhance our understanding of the pros and cons of various PDAC mouse models.

The second challenge of the TME dissection in response to therapies is cellular plasticity. The cell phenotype in the TME reflects a sum of stimuli (cytokines, chemokines, metabolites, growth factors, ECM molecules, neurotransmitters, etc.). A cascade of changes caused by therapy administration reprograms cells and affects the physiochemical property of the TME. Therefore, dissection of the TME in the context of therapy resistance is labor-intensive due to the TME’s sensitivity requiring precise experimental consistency. 

The third challenge is to balance the efficacy and toxicity when targeting the TME. Unlike tumor cells, cells of the TME lack common genetic alternations that can distinguish them from normal cells, and they are not exclusively present in tumors. It is difficult to specifically target tumor-associated cells without affecting the same cell populations in normal tissue. Thus, instead of attempting to eliminate pro-tumor cells (e.g., CAFs, TANs, TAMs), reprogramming tumorigenic cell populations into tumoricidal phenotypes seems more attractive. However, blockage of CSF1R using small molecule compound PLX3397 with immunotherapy in multiple malignancies (NCT02452424) or CSF1R neutralizing antibody Cabiralizumab (NCT03336216) with chemotherapy did not show improved efficacy in PDAC patients [153,154]. TME characterization is required to understand the failure, and additional targets for TME reprogramming need to be further explored.

In summary, accumulated scientific evidence highlights the importance of pancreatic TME in regulating therapy response and tumor recurrence. The development of novel KRAS targeted therapies brings new hope for PDAC patients [5], even though the mechanisms of TME regulation of therapy responses are not fully understood. In addition, further investigation is required to determine how to sensitize pancreatic tumors to immune checkpoint blockade. Benefiting from the single-cell assays, underrepresented cell types in TME can be characterized and examined whether they involve in therapy resistance. Breakthroughs in these areas will fundamentally change the current state of PDAC treatment.

## Figures and Tables

**Table 1:** Summary of therapy resistance mechanisms driven by tumor microenvironment in pancreatic cancer.

Cell Type	Therapy	Resistance Inducer	Detailed Mechanism	Reference
PSCs	chemotherapy (gemcitabine)	Collagen I	Promote proliferation by MAPK pathway activation and chromatin remodeling	[62,63]
		Periostin	Induce ECM molecules, including collagen I	[60,61]
		fibronectin	Promote proliferation by MAPK pathway activation	[64]
		IGF1, IGF2	Activate IGFR-PI3K-AKT pathway	[65]
		LIF	Activate Wnt and Hippo signaling pathways and induce EMT	[66]
		HGF	Activate c-Met-PI3K-Akt pathway and induce EMT	[67]
		CYR61	Downregulate nucleoside transporters ENT1 and CNT3	[68]
		Deoxycytidine	Compete with gemcitabine for deoxycytidine kinase-mediated phosphorylation	[69]
	immunotherapy	CXCL12	Chemoattract CD8+ T cells via CXCL12-CXCR4 axis to sequester them in the panstromal compartment	[70]
		Galectin-1	Induce T cell apoptosis and Th2 differentiation	[71]
		IL-6	Promote MDSC differentiation via STAT3 activation and suppress T cell proliferation	[72]
CAFs	chemotherapy (gemcitabine)	5′-nucleotidases	Entrap active gemcitabine intracellularly via downregulation of Nt5c1A, Nt5c3	[75]
		Exosomes	Deliver SNAI1 and miR-146a to tumor cells via exosomes	[76]
		circFARP1	Enhance LIF expression and secretion	[77]
		TGF-β	Upregulate ATF4 in tumor cells to activate ABCC1 expression	[78]
		SDF-1	Form a reciprocal feedback loop with tumor cells via SDF-1/SATB-1 axis	[79]
		IL-6	Activate JAK-STAT3 signaling pathway	[80]
		CXCL12	Bind to CXCR4 to activate FAK, AKT, and ERK pathways	[81]
	chemotherapy (oxaliplatin)	IL-8	Upregulate UPK1A-AS1 to facilitate DNA repair	[82]
	chemotherapy (etoposide)	NO	Elevate IL-1β production in tumor cells	[83]
	targeted therapy (EGFRi erlotinib)	NRG-1	Activate ERBB3-AKT signaling pathway	[84,85]
	immunotherapy	ECM	Form a physical barrier to impede T cell-tumor cell contact	[86]
		ROS	Induce M2 TAM polarization	[87]
		/	Suppress immunogenic activities	[88]
		CXCL12	Exclude T cells from tumor region by binding to CXCR4	[89]
		PGE2	Induce expression of immune checkpoints on CD4+ and CD8+ T cells	[90]
		TSLP	Induce Th2 cell polarization through dendritic cell conditioning	[41]
	targeted therapy (GOT2i)	Pyruvate	Provide tumor cells with pyruvate to maintain redox balance	[91,92]
TAMs	chemotherapy (gemcitabine)	Deoxycytidine	Interfere the uptake and metabolism of gemcitabine	[93]
		Cytidine deaminase	Elevate cytidine deaminase expression in tumor cells to inactivate gemcitabine	[94]
	targeted therapy (KRASi)	TGFβ	Activate canonical SMAD3/4 pathway and promote EMT	[95]
	immunotherapy	Granulin	Induce fibrosis to prevent T cell infiltration	[96]
		Mincle	Ligate to SAP130 expressed by tumor cells to suppress cancer immunity	[97]
		RIP1	Regulate M2 TAM polarization	[98]
	radiotherapy, immunotherapy	/	n/a	[99,100]
TANs	chemotherapy (gemcitabine)	IL-6	Activate JAK-STAT3 signaling pathway	[80]
	immunotherapy	NETs	Cause tumor CD8+ T cell inactivation and spatial exclusion	[101]
	chemotherapy (FOLFIRINOX, gemcitabine, nab-paclitaxel), radiotherapy, immunotherapy	/	n/a	[102,103,104,105,106,107]

EGFRi, EGFR inhibitor; KRASi, KRAS inhibitor; n/a, not addressed.

**Table 2:** Summary of representative clinical trials targeting TME in pancreatic cancer.

Target	Agent	Combined Agent	Selected Clinical Trials
*ECM or membrane proteins*			
Hyaluronic acid	PEGPH20	Avelumab, chemotherapy, pembrolizumab	NCT03481920, NCT01453153, NCT01839487, NCT04058964
Plectin	ZB131		NCT05074472
Galectin-9	LYT-200		NCT04666688
CTLA-4	Zalifrelimab		NCT04827953
RARα/β	Am80		NCT05064618
*Receptors*			
IGF1R	MK-0646	Chemotherapy + TKI	NCT00769483
	Cixutumumab		NCT00617708
	AMG 479	Chemotherapy, radiotherapy, AMG 655	NCT00630552, NCT01298401, NCT00819169, NCT01231347
	Metformin	Everolimus, octreotide LAR	NCT01971034, NCT02431676
	MM-141	Chemotherapy	NCT02399137
HER3	Seribantumab		NCT04790695, NCT04383210
	HMBD-001		NCT05057013
HER2/3	Zenocutuzumab (MCLA-128)		NCT02912949
IL6R	Tocilizumab	Chemotherapy	NCT02767557, NCT04258150
	CNTO 328		NCT00841191
CXCR4	MB1707		NCT05465590
	Plerixafor	Cemiplimab	NCT03277209, NCT02179970
IL1RAP	CAN04	FOLFIRINOX	NCT04990037
TGFβR	PF-06952229		NCT03685591
	SHR-1701	Chemotherapy	NCT04624217
CSF1R	Cabiralizumab	Nivolumab, chemotherapy	NCT02526017, NCT03697564
	Pexidartinib	Durvalumab	NCT02777710
	IMC-CS4	Pembrolizumab, GVAX	NCT03153410
CXCR2	SX-682	Nivolumab	NCT04477343
*Enzymes*			
COX	Etodolac		NCT03838029
	Celecoxib	Chemotherapy, irinotecan, interferon α-2b, DC vaccine	NCT00198081, NCT00068432, NCT00177853, NCT01111591
RIPK1	GSK3145095		NCT03681951
*Cytokines, chemokines, or growth factors*			
LIF	MSC-1		NCT03490669
HGF	Ficlatuzumab		NCT03316599
CXCL12	Olaptesed pegol (NOX-A12)	Pembrolizumab	NCT03168139, NCT04901741
IL-6	Siltuximab	Spartalizumab	NCT04191421
IL-12	VG161	Nivolumab	NCT05162118
IL-15	ALT-803		NCT02559674
IL-1β	Canakinumab	Spartalizumab, nab-paclitaxel, gemcitabine	NCT04581343, NCT04229004
IL-2	Aldesleukin	chemotherapy, anti-KRAS G12D mTCR PBL, anti-KRAS G12V mTCR PBL, pembrolizumab, anti-hCD70 CAR-transduced PBL, HER2Bi-armed T cells, sargramostim, ALVAC-CEA vaccine, neoantigen-specific TCR-T	NCT05194735, NCT02620865, NCT01583686, NCT01212887, NCT03745326, NCT01174121, NCT03190941, NCT02830724, NCT02662348, NCT00003125, NCT05194735, NCT04426669
IL-8	BMS-986253	Nivolumab	NCT02451982
VEGF	Bevacizumab	Chemotherapy, radiotherapy, TKI, cetuximab, ALT-803, cancer vaccine, immunotherapy, pembrolizumab, ZN-c3, PEGPH20, durvalumab, TGR-1202	NCT00047710, NCT00417976, NCT00614653, NCT00460174, NCT00365144, NCT00602602, NCT00410774, NCT00126633
	Bevacizumab-800CW		NCT02743975
	Avastin	Chemotherapy, NANT-008, radiotherapy	NCT03127124, NCT00735306, NCT00609765
	rhuMAB-VEGF	Chemotherapy	NCT00066677
TGF-β	HCW9218		NCT05304936
	BCA101		NCT04429542
	NIS793	PDR001, chemotherapy	NCT02947165, NCT05417386
	AP 12009		NCT00844064
M-CSF	MCS110	Spartalizumab	NCT02807844
GM-CSF	Sargramostim	Carcinoembryonic antigen peptide 1-6D	NCT00669734, NCT00012246
	GM-CSF	iNeo-Vac-P01, TG-01	NCT04810910, NCT03645148
	OH2 injection		NCT04637698
	PANC 10.05 pcDNA-1/GM-Neo		NCT01088789
	PANVAC™-VF		NCT00088660

TKI, tyrosine kinase inhibitor; PBL, peripheral blood lymphocyte.

## Data Availability

Not applicable.
